# Survivin-Based Treatment Strategies for Squamous Cell Carcinoma

**DOI:** 10.3390/ijms19040971

**Published:** 2018-03-24

**Authors:** Andrea Santarelli, Marco Mascitti, Lucio Lo Russo, Davide Sartini, Giuseppe Troiano, Monica Emanuelli, Lorenzo Lo Muzio

**Affiliations:** 1 Department of Clinical Specialistic and Dental Sciences, Marche Polytechnic University, Via Tronto 10, 60126 Ancona, Italy; marcomascitti86@hotmail.it (M.M.); davide_sartini@libero.it (D.S.); m.emanuelli@univpm.it (M.E.); 2 Dentistry Clinic, National Institute of Health and Science of Aging, INRCA, 60126 Ancona, Italy; 3 Department of Clinical and Experimental Medicine, Foggia University, Via Rovelli 50, 71122 Foggia, Italy; lorenzo.lomuzio@unifg.it (L.L.R.); giuseppe.troiano@unifg.it (G.T.); lorenzo.lomuzio@unifg.it (L.L.M.)

**Keywords:** survivin, squamous cell carcinoma, inhibitor of apoptosis, oral squamous cell carcinoma

## Abstract

Survivin, an anti-apoptotic molecule abundantly expressed in most human neoplasms, has been reported to contribute to cancer initiation and drug resistance in a wide variety of human tumors. Efficient downregulation of survivin can sensitize tumor cells to various therapeutic interventions, generating considerable efforts in its validation as a new target in cancer therapy. This review thoroughly analyzes up-to-date information on the potential of survivin as a therapeutic target for new anticancer treatments. The literature dealing with the therapeutic targeting of survivin will be reviewed, discussing specifically squamous cell carcinomas (SCCs), and with emphasis on the last clinical trials. This review gives insight into the recent developments undertaken in validating various treatment strategies that target survivin in SCCs and analyze the translational possibility, identifying those strategies that seem to be the closest to being incorporated into clinical practice. The most recent developments, such as dominant-negative survivin mutants, RNA interference, anti-sense oligonucleotides, small-molecule inhibitors, and peptide-based immunotherapy, seem to be helpful for effectively downregulating survivin expression and reducing tumor growth potential, increasing the apoptotic rate, and sensitizing tumor cells to chemo- and radiotherapy. However, selective and efficient targeting of survivin in clinical trials still poses a major challenge.

## 1. Introduction

### 1.1. Squamous Cell Carcinoma

The term “squamous cell carcinoma” (SCC) is used to indicate a heterogeneous group of different epithelial malignancies that arise from uncontrolled growth of epithelial cells [1]. These tumors typically develop in organs that are present in various parts of the body, which are united by the fact that they are covered with squamous epithelium [2]. The anatomical sites involved include the skin, oral cavity and oropharynx, larynx, esophagus, lungs, and genitourinary tract. Epidemiologically, most SCC cases are included in four categories: head and neck squamous cell carcinoma (HNSCC), esophageal cancer, non-melanoma skin cancer, and non-small cell lung cancer (NSCLC) [1,2]. Based on worldwide cancer data, SCCs are the group of tumors that are most commonly capable of metastasizing, representing a serious health problem [3,4].

Despite significant improvements in diagnostic procedures and therapeutic strategies, the mortality rates from SCC have remained generally high over the last decades. Non-melanoma skin cancer is one of the most common cancers worldwide, particularly affecting Caucasians [5]. This tumor in characterized by uncontrolled growth of abnormal keratinocytes and includes SCC and basal cell carcinoma (BCC). BCC is a relatively benign tumor, while SCC shows a higher mortality rate due to the increased risk of metastasis, although significantly lower than the other SCCs [2,6]. For this reason, lesion removal is the treatment of choice; chemotherapy has an important role in advanced/metastatic disease [6].

HNSCC is the sixth most common cancer worldwide. It is divided into several types according to anatomic location: oral, oropharyngeal, laryngeal, and nasopharyngeal [3,7]. The 5-year survival rate is below 50%, mainly due to late diagnosis. In fact, the treatment of choice for early stage HNSCC is surgical therapy with an overall 5-year survival rate of 75% [8]; multimodal treatment for locally advanced/metastatic HNSCC has failed to significantly improve the prognosis [2,3].

Esophageal cancer is the eighth most prevalent cancer in the world and the SCC variant is the predominant histological variant. Despite diagnostic and therapeutic advances, the prognosis is poor, mainly due to late diagnosis, biological aggressiveness, and ineffective treatment strategies [9]. In fact, complete response is rarely achieved and the 5-year survival rate is below 40% [10].

NSCLC accounts for approximately 85% of all cases of lung cancer and SCC type is one of the histological variants [11]. The SCC variant of NSCLC is typically present as the central lung tumor. The prognosis is very poor with a 5-year life expectancy in only 17% of patients with a newly diagnosed tumor [12].

One reason is that SCCs are often diagnosed at advanced stages because of the lack of reliable and early diagnostic biomarkers [13]. Thus, the identification of molecular markers for early detection and effective treatment of SCC are valuable and necessary.

SCC carcinogenesis is a complex multistep process involving the accumulation of several genetic alterations. These alterations promote progression from normal epithelial cells to clinically evident cancerous lesions capable of producing metastases [14]. Modern high-throughput methods in genomics, epigenetics, and molecular biology produce large amounts of data that can be useful in both discovery of new gene abnormality patterns of SCC and in the identification of new prognostic biomarkers [2].

Many molecular markers have been discovered in SCCs and several important similarities have been found among the major groups of SCCs, including abnormalities in cell cycle regulatory proteins (p53 family and Ki-67) and signal transduction proteins (EGFR).

p53 is a tumor suppressor protein with a crucial anti-cancer role that promotes cell cycle arrest, senescence, and apoptosis in response to stress signals. p53-inactivating mutations have been found in more than 50% of all human cancers, including SCCs [15]. Mutations in p53 is a critical step in the development on non-melanoma skin cancer [16] and is also commonly observed in HNSCC [17], esophageal cancer [18], and NSCLC [19]. Although p53-inactivating alterations are present in the vast majority of SCCs, currently it is considered a prognostic factor only for HNSCC and esophageal cancer [2]. Another member of the p53 family is p63, which plays a critical role in regulating self-renewal of epithelial tissues [20]. p63 is considered a prognostic factor for SCC, although with different significance. In fact, p63 is highly expressed in non-melanoma skin cancers, while it is considered a positive prognostic factor in HNSCC and NSCLC [2]. The reason is that p63 includes different isoforms, which have different roles in carcinogenesis of SCC [20]. EGFR, a member of the ErbB family protein of receptor tyrosine kinases, is a trans-membrane cell surface receptor that has an important role in fetal development, including the epithelial tissues. EGFR overexpression is found in the vast majority of HNSCC [21] and is considered a negative prognostic factor in all the major groups of SCCs [2].

The altered functions of the cell cycle regulatory proteins commonly found in SCCs suggest that studies of programmed cell death processes can lead to new strategies for the treatment of these tumors.

### 1.2. Apoptosis, Survivin, and Tumorigenesis

Human cancers are thought to be characterized by an imbalance in the cellular and tissue regulatory mechanisms involved in cell cycle progression and apoptosis. The term “apoptosis” refers to the most studied form of programmed cell death. It is described as a physiological process that plays a role in removing senescent, unneeded, or dangerous cells that sustain DNA damage to undergo uncontrolled cellular proliferation. Any alteration in this sophisticated molecular mechanism can cause abnormal inhibition of cell death and tumorigenesis, as well as an increase in chemoresistance.

Almost 25 years ago, a protein family named inhibitor of apoptosis (IAP) proteins was discovered that encodes a group of proteins such as X-linked inhibitor of apoptosis (XIAP), c-IAP, and survivin [22]. These proteins are evolutionarily conserved from viruses to mammals [22,23] and several studies have found high levels of these proteins in different types of human cancers [24,25]. IAP proteins negatively regulate apoptosis through the inhibition of caspase activity [26,27]. Caspases are a well-known group of proteolytic proteins that are responsible for cleavage of other proteins, leading to apoptosis [28,29]. The most important functions of IAPs are direct inhibition of the terminal effector caspase-3, -6, and -7 [30,31], and interference with the intrinsic caspase cascade by blocking caspase-9. Other functions exerted by IAPs involve cell cycle regulation, ubiquitination, and proteasome-mediated protein degradation [26,27,32].

Survivin, an IAP protein discovered about 20 years ago, is highly expressed in several human malignancies but also in normal adult cells. In fact, survivin was detected for the first time in normal adult thymus and placenta [33]; subsequent studies revealed the presence of this protein in many adult tissues, although the levels found were lower than in tumor cells. More sensitive methods have revealed the expression of survivin in primitive hematopoietic cells [34,35], T lymphocytes [36], polymorphonuclear cells [37], vascular endothelial cells [38,39], keratinocytes [40], testes [41], and ovary [42]. Some studies demonstrated significant expression of survivin in endothelial cells during the proliferative phase of angiogenesis, suggesting a direct role in tumor angiogenesis and carcinogenesis [38,39,43].

**Figure 1:**
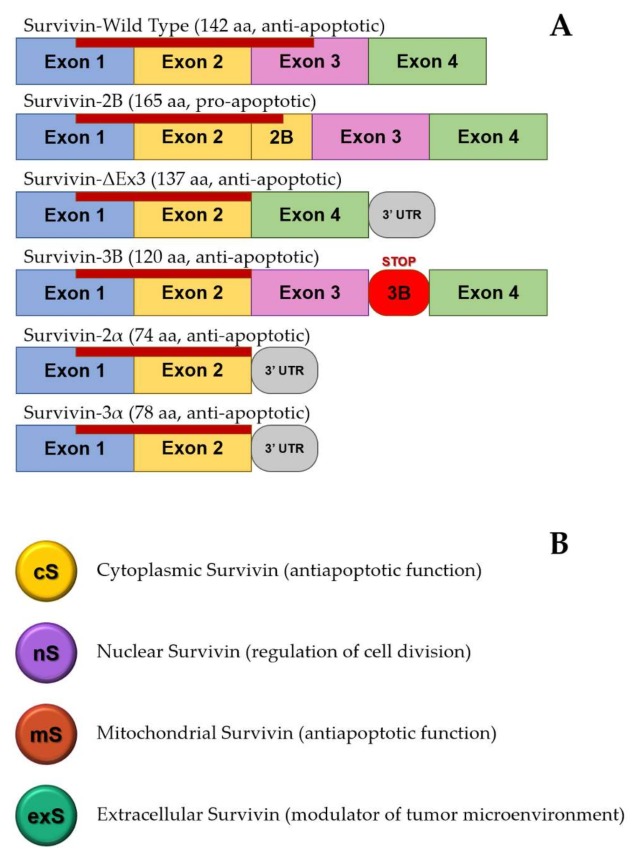
Different isoforms and subcellular localizations of survivin that provide functional heterogeneity. (**A**) Survivin splice variants described to date. For each variant, the number of amino acids and the effects on programmed cell death are reported. Survivin-2B retains part of intron 2. Survivin-3B possesses an additional 3B exon with a stop codon. Baculovirus IAP repeat (BIR) domain position is reported as a red line. (**B**) Subcellular localization and main functions of survivin.

Five alternative splice variants of survivin (survivin “wild-type”, survivin 2α, survivin-β/2B, survivin-3B, and survivin-ΔEx3) have been identified [44,45,46,47,48,49], each one probably characterized by a different apoptotic function and cell localization (Figure 1). The function of survivin-3B (possessing an additional 3B exon with a stop codon) is not well defined, while functional assays showed that survivin-2α attenuates the anti-apoptotic activity of survivin. Survivin-2B (retaining part of intron 2 as a cryptic exon) is devoid of anti-apoptotic potential and may act as an antagonist of the anti-apoptotic survivin. Survivin-ΔEx3 lacks 118 base pairs of exon 3. It is still unclear whether alternative splicing variants of survivin are the consequence of tumor adaptation used to support their proliferation and avoid detection by the immune system, and further investigations are needed [50].

Some investigators have suggested that survivin is linked to multiple pathways of cellular homeostasis, playing a key role in many cellular functions. In fact, this protein has multiple functions regarding the regulation of cell division, the modulation of apoptotic and non-apoptotic cell death, the maintenance of cell proliferation and survival in unfavorable conditions, and the resistance to various anticancer therapies [51]. Some authors suggested that the primary function of survivin is to regulate cell division rather than inhibit apoptosis [52,53]. In fact, survivin levels increase in the G1 phase, reaching a peak during the G2M phase of the cell cycle. Furthermore, during the cell cycle, survivin exists within a multi-protein complex known as the chromosomal passenger complex (CPC) [54,55,56], contributing to chromatin-associated spindle formation [57].

Recently, survivin has been identified also in extracellular vesicles from biological fluids [58]. These cell-derived vesicles, called exosomes, are constitutively released by different cell sources, including cancer cells [59]. The exosome-related survivin, or more simply “extracellular survivin”, provides a protective role to the neighboring cancer cells, modulating the tumor microenvironment [60]. Extracellular survivin, as well as the exosomes, may also be used as a tool for the early detection of malignant conditions, improving the diagnostic performance of the so-called “liquid biopsy” that is already evaluated for many biological fluids [61,62,63].

**Figure 2:**
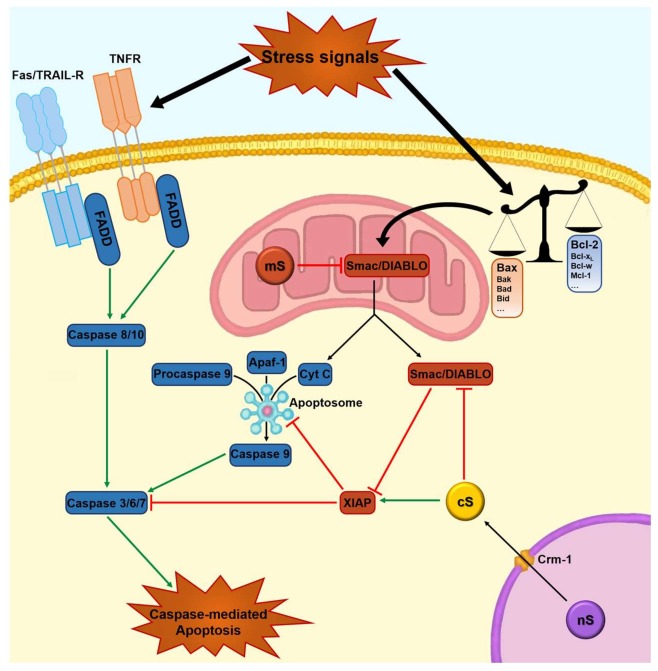
Role of survivin in caspase-mediated apoptosis. The activation of caspase-3 and caspase-mediated apoptosis are caused by the initiator caspases (caspase-8/-10 and -9) as part of the extrinsic and intrinsic apoptotic signaling pathways, respectively. In the extrinsic pathway, activated receptors, such as Fas and tumor necrosis factor receptor (TNFR), cause caspase-8/-10 activation. In the intrinsic pathway, stress signals alter the balance of pro- and anti-apoptotic members of the Bcl-2 family, resulting in the release of mitochondrial factors such as Smac/DIABLO and cytochrome c. These factors cause the activation of caspase-9. Survivin interacts with XIAP, preventing the activation of caspase-9 through the inhibition of apoptosome formation and with Smac/DIABLO hinders its pro-apoptotic function. The nuclear/cytosolic balance of survivin is regulated by the nuclear export receptor Crm1. Green-line arrow: stimulation; red t-bar: inhibition; black-line arrow: translocation of molecules/stimuli.

This checkpoint function is linked to other pathways in which survivin regulates apoptosis. The activation of the checkpoint kinase Checkpoint Kinase 2 (CHK2) by DNA damage determines the rapid expulsion of the mitochondrial pool of survivin into the cytosol, antagonizing DNA damage-induced apoptosis. This pathway is antagonized by a parallel survivin-p53 subsystem in which the stabilization of p53 due to DNA damage allows this protein to function as a repressor of survivin transcription, lowering its levels [51,64]. The role of survivin in blocking apoptosis through direct and indirect inhibition of the activity of caspases-3, -6, -7, and -9 is well known [27,64]; it therefore plays a pivotal role in cell survival [65] (Figure 2). Other lines of evidence have demonstrated that mitochondrial survivin, localized in the inter-mitochondrial membrane space, has an autonomous anti-apoptotic function, although the underlying molecular mechanisms are still enigmatic [66,67]. Interestingly, mitochondrial survivin is undetectable in normal cells, suggesting that this specific subset may be related to oncogenic transformation [1].

Unlike normal cells, constitutively activated nuclear factor-kappa B (NF-κB) is detected in a wide variety of human malignancies and several studies showed upregulation of survivin expression at a transcriptional level by NF-κB, which in turn can be activated by growth factors via the phosphatidylinositol 3-kinase (PI3K)/Akt pathway and by TCF-4/β-catenin pathway [64,68,69]. The regulation of survivin expression can also be performed through other pathways, as demonstrated by Carter et al. [70] in acute myeloid leukemia. The results showed that the expression of this protein is upregulated by hematopoietic cytokines such as GM-CSF, G-CSF, and SCF through MEK/ERK and Phosphatidylinositol-3-kinases (PI3K) signal transduction pathways. STAT3, another transcription factor involved in cancer development, is constitutively activated in cancer cells from various tissues [71,72] and the results of several studies suggest a direct contribution of STAT3 to malignant progression through survivin signaling.

Survivin is degraded by the ubiquitin-proteasome pathway during the G1 phase of the cell cycle, while it is stabilized when bound to heat shock protein 90 (Hsp90), a member of the Hsp family [73]. Survivin is inhibited by Smac/DIABLO, a mitochondrial protein that binds IAPs, allowing the caspase cascade to progress and to activate apoptosis [74,75].

**Figure 3:**
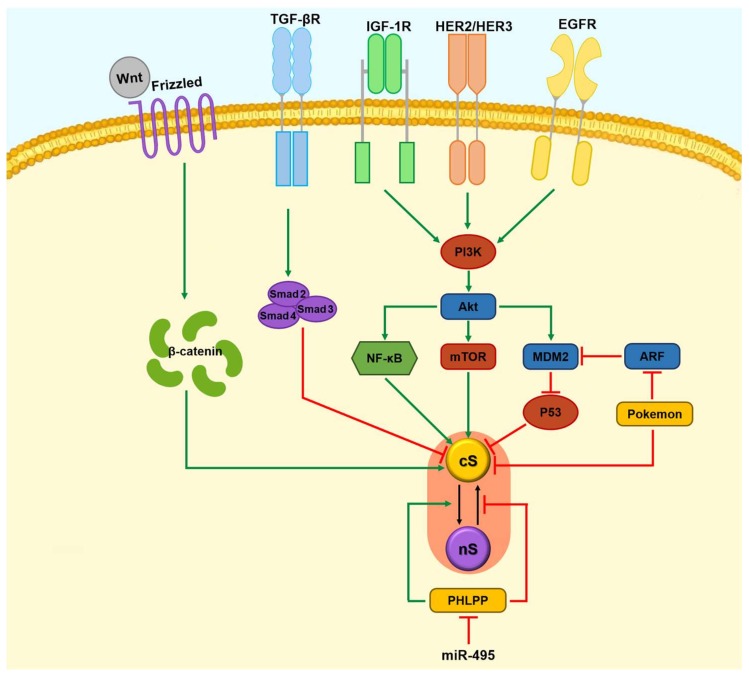
Schematic diagram of survivin regulatory patterns. Survivin expression is associated with aberrant activation of several tyrosine kinase receptors. PI3K-Akt-mTOR pathway and some upstream regulatory receptors (EGFR, HER2, HER3, and IGF-1R) play a key role in survivin homeostasis by upregulating its expression and counteracting the effects of repressors such as p53. Activation of Wnt signaling pathway leads to cytosolic accumulation of β-catenin, which then translocates to the nucleus and upregulates survivin. The Transforming Growth Factor (TGF) signaling pathway downregulates survivin expression through a Smad-dependent mechanism. Among the regulatory patterns that influence survivin expression, Pokemon seems to activate survivin signaling by suppressing the p53 pathway. Furthermore, the nuclear/cytosolic balance of survivin can also be regulated by other molecules, such as miR-495/PHLPP pathway. Green-line arrow: stimulation; red t-bar: inhibition; black-line arrow: translocation of molecules. cS = cytosolic survivin, nS = nuclear survivin.

Recent studies found that other pathways influence the expression of survivin by acting on the transcriptional level or by altering nuclear/cytosolic balance of survivin, suggesting the presence of a complex regulation system that has not yet been completely clarified [64,76,77] (Figure 3).

Interference with survivin functions induces programmed cell death and cell division defects [78], suggesting a dual role of this protein in apoptosis control and mitotic cell division. Survivin expression is correlated with poor prognosis, increased local recurrence rates, and reduced overall survival time in many cancers [79,80,81,82,83]. In model cancer cell types, overexpression of survivin was responsible for a cytoprotective effect, counteracting apoptosis induced by Fas/TNF-R (tumor necrosis factor receptor) engagement, pro-apoptotic proteins such as Bax, effector caspases, and several chemotherapeutic drugs [31] (Figure 2). These features are consistent with a critical role of apoptosis inhibition in cancer progression.

The increased expression of survivin appears to be associated with the grade of metastasis and aggressiveness, reduced survival, and an increased risk of resistance to therapy in many tumor types [23,84,85], suggesting a role as an unfavorable prognostic marker [86]. Some authors reported that survivin stimulates adenomas with mild dysplasia into highly dysplastic lesions, playing an important role in colorectal tumorigenesis. In fact, dysplastic transition is associated with a reduced apoptosis index [87]. In patients with malignant melanoma, breast, esophageal, and prostate cancer, survivin expression is positively modulated and very often correlated with poor response to anticancer therapy, disease recurrence, and decreased overall survival [88,89,90,91]. Regarding oral squamous cell carcinoma (OSCC), in our previous studies we found that survivin is expressed in about 80% of this tumor type, and that its degree of expression is directly related to an aggressive phenotype [83,85,92,93]. Indeed, OSCC patients with higher survivin expression show worse prognosis than those with lower survivin expression [94]. Moreover, survivin overexpression was found to be an early event of malignant transformation in precancerous and cancerous lesions of the oral cavity, as survivin was expressed in 94% of oral precancerous lesions that evolved into OSCC [95].

Results from human transcriptome analysis showed that survivin is the fourth most highly expressed protein in cancer tissue compared with normal counterparts [96,97]. The elevated expression of survivin in cancer tissues has prognostic and therapeutic implications. Indeed, from the point of view of anticancer therapy, the multiple functions performed by survivin in molecular processes that are typically altered in cancer cells suggest that targeting survivin may be advantageous compared with targeting other molecules involved in a single oncogenic pathway [98]. In fact, this protein intersects multiple cellular network and disabling it can compromise multiple signaling networks required for tumor cell maintenance, bypassing the ability to elicit resistance through mutations by cancer cells [99]. Furthermore, survivin expression is regulated by developmental signaling pathways that sustain stem cells, suggesting the possibility of using survivin antagonists with the aim of affecting cancer stem cells [100]. Another advantage is that this protein is highly expressed in endothelial cells during the proliferative phase of angiogenesis; therefore, it has a direct role in tumor angiogenesis and carcinogenesis [38,39,43]. In vitro studies showed that survivin inhibitors act on both cancer cells and endothelial cells in the tumor mass.

Since most cytotoxic agents currently in use induce apoptosis of cancer cells and survivin upregulation seems to play a pivotal role in resistance to chemo- and radiotherapy [24,101], new classes of targeted drugs are emerging based on strategies to inhibit the expression or function of survivin.

## 2. Gene Therapy

### 2.1. Dominant-Negative Survivin Mutants

Recently, the promise of gene therapy began to be realized in preclinical investigations and, even more recently, in clinical trials. The idea that prompted research in anticancer gene therapy is that this approach has several appealing aspects, particularly in those tumors with specific genetic alterations in which conventional treatment modalities have been largely unsuccessful. Among the gene therapy approaches targeting survivin, plasmids or viral vectors have been successfully used to deliver dominant-negative survivin mutants to tumor cells.

Grossman et al. [102] first demonstrated that transfecting a model keratinocyte cell line, HaCat cells, with a green fluorescent protein-conjugated survivin dominant negative mutant carrying a cysteine 84/alanine (Cys84Ala) mutation in the survivin baculovirus IAP repeat domain resulted in spontaneous apoptosis with a five-fold increase in the sub-G0/G1 fraction, corresponding to apoptotic cells. The effectiveness of the survivin mutant (Cys84Ala) was also demonstrated in melanoma [103], colon [104], and gastric [105] cancer cell lines.

Active forms of survivin result after phosphorylation of its threonine 34 (Thr34) by the cyclin-dependent kinase p34-cyclin B1 protein complex [35]. Loss of Thr34 phosphorylation produces dissociation from the caspase-9-survivin protein complex, resulting in caspase-dependent cell death [106,107,108]. The phosphorylation-defective Thr34/alanine (Thr34/Ala, or T34A) mutant is the most studied survivin mutation, suggesting its role in promoting sensitivity to chemo- and radiotherapy through apoptosis enhancement [109]. Recently, Barrett et al. [110] showed that modification of T34 in the HeLa cell line has both a positive and a negative impact on survivin function. In fact, cancer cells harboring survivin T34A mutant forms are not protected against death-inducing signals, but show growth acceleration.

In contrast, the phosphomimetic form of T34 (T34E) retards mitosis and it significantly enhances cell resistance to death-inducing signals. Therefore, survivin-T34A is dominant in proliferation and dominant negative during apoptosis, while survivin-T34E demonstrates opposite biological features. Results from several studies investigating survivin-T34A in cancer cells and xenograft models suggest that the role of this mutant form is to promote sensitivity to chemo- and radiotherapy through apoptosis enhancement.

### 2.2. RNAi (Small Interfering RNAs, Short Hairpin RNAs, and MicroRNAs)

The demonstration that the siRNA approach is able to repress survivin was first provided in a SCC line (HeLa, cervical carcinoma). Indeed, Carvalho et al. showed that survivin was undetectable in cell cultures 60 h after transfection with specific siRNAs [111]. Subsequently, many different studies have highlighted that it is possible to reduce the proliferative potential in different human tumor cells through survivin knockdown by using siRNAs or plasmid/viral vectors encoding for short hairpin RNAs (shRNAs) [112,113]. Moreover, survivin downregulation was able to inhibit the tumor cell growth in nude mice xenografts [112,113] and reestablish the apoptotic response to pharmacologic treatments in different cancer cell types.

The delivery of siRNA to OSCC cells downregulated the expression of survivin, resulting in proliferation inhibition and apoptosis enhancement. Moreover, the cytotoxicity of chemotherapeutic agents such as cisplatin and 5-fluorouracil (5-FU) was enhanced even at low doses [114]. The same results were achieved by transfecting a plasmid-containing survivin shRNA into an OSCC tongue cell line: shRNA inhibited the expression of survivin at both the protein and mRNA level, inhibited cell proliferation via caspase-3 activity induction, and enhanced chemosensitivity to cisplatin [115]. Survivin shRNA-mediated knockdown suppressed tumor growth and induced apoptosis after transfection into human laryngeal carcinoma cell lines [116]. Ngan et al. [117] evaluated the responses to oxaliplatin, a third-generation platinum-based chemotherapeutic agent, in two esophageal cancer cell lines, one derived from SCC and one from adenocarcinoma. They found that transfection of siRNA targeted against survivin strongly inhibited the protein expression, resulting in a “mitotic catastrophe” in both cell lines. This confirmed the results of Wang et al. in which a plasmid vector encoding siRNA efficiently and stably suppressed survivin expression in another esophageal SCC cell line, leading to an increase in the apoptotic rate and inhibition of tumor cell growth both in vitro and in nude mice xenografts [118]. The plasmids, widely used in the abovementioned works, are non-viral vectors that show some interesting and potentially useful features, such as the dimension of the insert size and immunogenicity. However, in vivo transfection is not as efficient as that of viral vectors, representing their major limitation. The ability of RNAi-mediated survivin knockdown to reduce the formation of new tumors and to hinder the growth of already established lesions in vivo was confirmed in BALB/c nude mice bearing OSCC treated with a lentiviral vector encoding shRNA targeting survivin [113]. Moreover, the apoptotic rate induced using vincristine was increased as well as the susceptibility to Adriamycin of OSCC cells [113]. Adenovirus-based vectors are the most widely employed method for in vivo gene therapy. Other viral vectors, including adeno-associated virus, vaccinia virus, and lentivirus, have been extensively tested in preclinical studies [119], but clinical trials are still few [120].

The effects of survivin-based RNAi have also been studied in HNSCC cell lines, showing increased sensitivity to paclitaxel [121].

The discovery that a family of small noncoding RNA called microRNA (miRNA)—which are complementary to the 3′ UTR of the target mRNA—can negatively regulate target genes has led to the possibility of using them in cancer-specific gene therapy [122,123]. Currently, there are several studies addressing the use of miRNAs as anticancer therapy; some have evaluated the possibility of silencing survivin gene expression, inducing apoptosis, and inhibiting cell growth, also in the HeLa cell line [124,125].

### 2.3. Anti-Sense Oligonucleotides

The survivin anti-sense oligonucleotides (ASO) approach was first introduced by Grossman et al. [103] to trigger spontaneous apoptosis in the absence of other death-inducing signals in melanoma cell lines. Successively, other works have highlighted that it is possible to reduce the proliferative potential by inducing caspase-dependent apoptosis in different human tumor cells through specific inhibition of survivin mRNA and protein using chemically synthesized ASO [112]. Moreover, survivin downregulation was able to inhibit tumor cell growth in nude mice xenografts [126] and to reestablish the apoptotic response in cancer cells of different histologic origin to pharmacological and radiation treatments [127].

In a recent work, Sah et al. [128] showed that targeting survivin by incubating human epidermoid carcinoma cell line with a specific ASO for 24 h determined a dose-dependent inhibition of protein expression. Moreover, the suppression of survivin by ASO treatment determined radiosensitizing effects, even if it was probably related to effects on DNA repair rather than to real enhancement of apoptosis. However, a report concerning the ASO-mediated downregulation of survivin in HNSCCs demonstrated the enhancement of cisplatin and etoposide efficacy. In fact, when used in association with ASO, chemotherapeutic agents enhanced their cytotoxic effects on human laryngeal carcinoma HeP2 and tongue carcinoma Cal27 cells through restoration of apoptosis in these SCC cell lines [129]. Enhancement of cisplatin sensitivity in HNSCC transfected with an adenoviral vector encoding survivin ASO was subsequently confirmed in both an SCC cell line and in vivo xenograft nude mice model [130].

A phase I study established tolerability of 750 mg of LY2181308, another ASO [131], followed by two other phase I trials in patients with advanced cancers. LY2181308 (ISIS 23722; Lilly and Co., Indianapolis, IN, USA and ISIS Pharmaceuticals, Carlsbad, CA, USA) is a novel 2′-*O*-methoxymethyl modified second-generation ASO designed to complementarily bind to human survivin mRNA, with the aim of inhibiting its expression and restoring the physiological programmed cell death pathway in cancer cells [132]. Indeed, it was established that the anti-neoplastic activity of LY2181308 is related to survivin inhibition [133,134] and reduced expression of survivin mRNA and protein in tumor cell lines [78]. In the first trial, Talbot et al. [131] treated 40 patients with LY2181308 at doses ranging from 100 to 1000 mg; the patients had different tumors including breast, colon, and melanoma. In this dose-escalation study, the ASO was administered intravenously as 3-h intravenous loading doses on three consecutive days and followed by weekly maintenance doses. Results were promising with a total of 26 patients having the recommended dose of 750 mg, an acceptable safety profile, and manageable adverse events. Fever, fatigue, nausea, and elevated partial thromboplastin times were common side effects, while headache was experienced at the highest dose tested (1000 mg). Consistent with other second-generation ASOs and with preclinical models, pharmacokinetic profiles showed rapid clearance, rapid tissue distribution, and terminal half-life of 31 days. The major results of the study were the demonstration that ASO accumulates within tumor tissues, reduces survivin gene and protein expression by 20%, and restores apoptotic signaling in tumor cells in vivo. However, the clinical response was modest, obtaining stable disease in only 10% of patients.

In the second phase I trial, the tolerability, pharmacokinetics, and anti-cancer activity of LY2181308 were evaluated in 14 patients with advanced cancers [132]. In this dose-escalation study there was also one patient with esophageal SCC who was enrolled and treated with the recommended dose of 750 mg. The survivin ASO also demonstrated a favorable toxicity profile in this trial, showing no significant differences between ethnicities. The most common toxicities found were elevated PT-INR, thrombocytopenia, fatigue, and flu-like symptoms with fever being present in all patients reaching the highest dosage (750 mg). The pharmacokinetic profile showed rapid tissue distribution and a half-life of 21 days. The limited clinical response was consistent with the findings of Talbot et al., displaying a stable disease in only one patient with intrahepatic cholangiocarcinoma while the others had progressive disease [131]. Despite the data on the clinical response, the results from both trials showing favorable toxicity and pharmacokinetic profiles of LY2181308 have to be considered as positives. In fact, LY2181308 was not designed as a single-agent therapy; it was expected to facilitate the effects of pro-apoptotic treatments and thus have activity in conjunction with apoptosis-inducing agents, such as chemo- and/or radiotherapy.

## 3. Pharmacologic Therapy

### Small-Molecule Inhibitors

In the context of a strategy focused on pharmacologic inhibition of survivin, small molecules that directly or indirectly affect survivin have been developed and are currently under preclinical and clinical investigations.

1-(2-Methoxyethyl)-2-methyl-4,9-dioxo-3-(pyrazin-2-ylmethyl)-4,9-dihydro-1*H*-naphtho[2-d]imidazolium bromide (YM155) (Astellas Pharma, Inc., Deerfield, IL, USA) is a novel small-molecule inhibitor (SMI) that has been shown to selectively suppress survivin gene transcription and protein expression in vitro [112]. This molecule showed the capacity to inhibit the growth of cancer cell lines derived from hormone-refractory prostate cancer [135], NSCLC [136], non-Hodgkin’s lymphoma, ovarian cancer, sarcoma, breast cancer, leukemia, and melanoma [137], being active in the nanomolar range. Moreover, this small imidazolium-based compound exhibited potent anticancer activity in nude mice human hormone-refractory prostate [135], NSCLC, melanoma, breast cancer, and bladder cancer xenografts [138]. Iwasa et al. showed that treatment with YM155 resulted also in the ability to sensitize NSCLC cells to radiation [136] and to platinum-based compounds (cisplatin and carboplatin) [139] both in vitro and in vivo. These preclinical results and pharmacokinetic evaluation demonstrated a rapid elimination half-life in mice, rapid distribution to tissues, and a 20-fold increase in tumor tissue concentrations as compared with plasma [140], leading to clinical development of YM155 with two phase I trials [140,141]. In the first trial, 41 patients with advanced solid malignancies or lymphoma, including a case of HNSCC, received cycles of YM155 at doses ranging from 1.8 to 6.0 mg/m^2^/day by 168-h continuous intravenous infusion every 3 weeks. The second trial was an open-label, single-center, nonrandomized study with 33 patients affected by advanced refractory solid tumors, including 6 patients with esophageal SCC, treated with escalating doses ranging from 1.8 to 10.6 mg/m^2^/day of YM155 administered in the same way as the first study. The most commonly reported adverse reactions were microalbuminuria, fever, injection-site phlebitis, fatigue, and a reduction in hemoglobin, blood albumin, and lymphocyte counts. Since 92.2% of drug-related adverse events were classified grade 1 or 2 in severity, YM155 administration was judged as being safe. Considering both studies in terms of clinical response, one complete and two partial responses were achieved in three patients with non-Hodgkin’s lymphoma and a stable disease was achieved in nine patients with solid tumors, but no patient with esophageal SCC.

Recently, two phase II trials evaluating YM155 have been completed: an open-label, multicenter trial consisting of administration of YM155 as a single-agent therapy in patients with unresectable stage III–IV melanoma [142] and a multicenter study of YM155 as a single-agent therapy in patients with refractory previously-treated, advanced NSCLC [143].

Other small-molecules that do not directly target survivin but affect its expression have been proposed. Cyclin-dependent kinase (CDK) inhibitors such as purvalanol A have been developed with the aim of blocking the phosphorylation of survivin on Thr34, leading to increased protein destruction and thereby counteracting its function [144]. Recently, Pennati et al. [145] reported that the addition of the novel CDK inhibitor NU6140 to paclitaxel-treated HeLa cervical carcinoma cells resulted in significantly increased cytotoxic effect and apoptotic response in comparison with the paclitaxel-purvalanol A combination, resulting in abrogation of the expression of the survivin active form.

Downregulation of survivin, coupled with changes in key gene expression, was also suggested to be the potential mechanism by which the histone deacetylase inhibitor LBH589 causes G2/M cell cycle arrest and cell death in HNSCC cell lines [146].

## 4. Anti-Tumor Immunotherapy

Anti-tumor immune therapy can be reached in three ways: vaccines, enhancement of the immune response, and specific immune therapy with biological drugs.

Vaccines can be divided into first generation (obtained by using lysates of tumor cells of whole autologous/allogeneic tumor) and generation (obtained by using tumor-specific antigens). The tumor antigens identified for this purpose are mainly those of the melanoma-associated antigen (MAGE) family, expressed in 10–70% of solid tumors, which are absent or poorly expressed in normal tissues with limited expression of MHC class I in tissues such as testis and placenta. These molecules represent excellent targets for T cells and/or antibodies. Other antigens are those proteins that are present in normal tissues but are expressed in modified form in tumor tissues, such as p53 (50% of solid tumors), RAS, β-catenin and CDK4 (melanoma), and caspase 8 (HNSCC). This difference between tumor and normal tissues is of primary importance because the immune response following the vaccine should be directed exclusively at the cells of the diseased tissue, saving as much as possible the healthy tissue.

Since survivin is abundantly present during the development phases and is expressed in most human neoplasms, but is undetectable in most adult differentiated normal tissues [97], it represents an ideal candidate for eliciting a T-cell immune response. Cytotoxic T lymphocytes (CTL) have an important role in cancer immune surveillance due to the ability to detect quantitative and qualitative antigenic differences in transformed cells [147]. Some studies found the in vivo and ex vivo presence of survivin-reactive antibodies [148] and CTL clones restricted to several haplotypes [149,150,151], pointing out the immunologic properties of this protein and suggesting that survivin could be an attractive target for novel immunotherapies against cancer [97,152].

A phase I clinical trial started in September 2003 with the aim of evaluating the effects of survivin-2B peptide vaccine therapy in patients with advanced or recurrent OSCC [153].

Among the different splicing variants, survivin-2B was used in clinical vaccine therapy phase I/II trials. In preclinical studies, Hirohashi et al. [154] reported that survivin-2B is abundantly expressed in several tumor cell lines and its derived peptide is recognized by CTLs that then recognize survivin-positive HLA-A24^+^ cancer cells. Recently, the same group showed that it is possible to induce, from peripheral blood mononuclear cells, survivin-2B peptide-specific CTLs in HLA-A24-positive patients with colorectal cancers and OSCC [155,156], and exert cytotoxicity against adenocarcinoma cells.

The only clinical trial dealing with SCC was conducted by Miyazaki et al. [153] on patients with advanced or recurrent OSCC. However, of the 11 patients (6 males and 5 females) enrolled in the study (one patient discontinued the treatment after four vaccinations), only seven were histologically SCC; the others were adenoid cystic carcinomas or alveolar soft part sarcoma. SCC patients received vaccination with survivin-2B80-88 peptide (amino acid sequence AYACNTSTL) derived from a splicing variant survivin-2B-specific exon 2B; vaccinations were administered subcutaneously into the ipsilateral neck or intratumoral six times at intervals of 14 days with two incremental dose levels: three patients received 0.1 mg and four patients 1.0 mg. The major result from this phase I trial was the absence of toxicity after peptide administration, even in patients who received the higher dose of 1.0 mg. No patients experienced skin reactions, hematological, cardiovascular, hepatic or renal toxicity during or after vaccination. However, despite the absence of adverse effects, only a marginal immunological and clinical response was achieved. Only in one case was a transient 2-month partial clinical response observed, achieving a tumor regression rate of 70% on CT imaging, disappearance of the neck metastatic tumor, and a 10 times lower serum SCC antigen level. In the remaining patients, disease progression was observed and the data about the tumor marker level used for monitoring the clinical response in these patients were difficult to interpret. In addition, considering two recent studies investigating the effectiveness of different serum tumor markers in 136 and 527 patients with HNSCC, respectively, only TPA-M and CYFRA 21-1 [157] and IL-6 [158] significantly improved outcome prediction.

### Adoptive Cell Therapy

The second route of anti-tumor immunotherapy involves the intravenous administration of tumor-specific autologous T cells expanded ex vivo, the so-called adoptive cell therapy. One of the major obstacles of this therapeutic strategy is the lack of long-term CTL persistence in the tumor-bearing host after transfer [159]. However, Rosenberg et al. [160] reported a 34% response rate among patients with metastatic melanoma who were treated with adoptive cell therapy, with the possibility to improve the response rate to 49% by adding lymphocyte-depleting chemotherapy before the administration of CTL [161,162]. Unfortunately, only a few investigations with survivin have been conducted and none in the SCC type.

## 5. Side Effects

Despite the essential role of survivin during development and in homeostatic function in certain adult tissues, its antagonists that have been tested were tolerated with modest side effects. Due to the multiplicity of functions of survivin, future therapies aimed at its inhibition might cause still unknown side effects. In fact, the absence of significant side effects on hematopoietic cells reported in the studies may be caused by the local intratumor administration and minimal systemic dissemination of anti-survivin therapies, which may not have given a true toxicity profile. To investigate the possible side effects, Leung et al. [163] abolished survivin expression from the hematopoietic compartment of mice, showing that inducible deletion causes defects in erythropoiesis in a subset of the animals, which emphasizes the sensibility of the hematopoietic system to survivin disruption. Regarding the effects of survivin on T lymphocytes, Sharief et al. [164] showed that survivin is upregulated in T lymphocytes from patients with multiple sclerosis, and treatment with interferon β-1a leads to the reduction in clinical exacerbations. This effect was mediated by downregulation of survivin in these cells, augmenting T-cell susceptibility to apoptosis. However, in agreement with Altieri [51], there is the possibility that targeting survivin might preferentially affect the qualitatively different molecular networks in cancer cells, leaving survivin functions intact in normal tissues. This would make survivin a nodal cancer protein, orchestrating potentially tumor-specific signaling networks, making its pathways preferentially operational in cancer cells. These hypotheses can alleviate concerns regarding possible serious side effects consequent to systemic survivin-based therapy, such as inhibition of T cell and hematopoietic progenitor proliferation, in which survivin plays an important homeostatic role.

## 6. Future Directions

Since inhibition of mechanisms underlying apoptosis events can promote tumorigenesis and increase resistance to chemo- and radiotherapy, induction of apoptosis has been considered a promising method for anticancer therapy. Survivin, a structurally unique IAP family member involved in the suppression of apoptosis, has been reported to contribute to carcinogenesis and drug resistance in a wide variety of human cancers. Furthermore, survivin is abundantly expressed in most human neoplasms but is undetectable, under physiologic conditions, in most adult differentiated normal tissues. These observations have generated considerable interest in developing new cancer therapies that target survivin with the aim of creating a synergistic effect with conventional cancer treatments.

**Figure 4:**
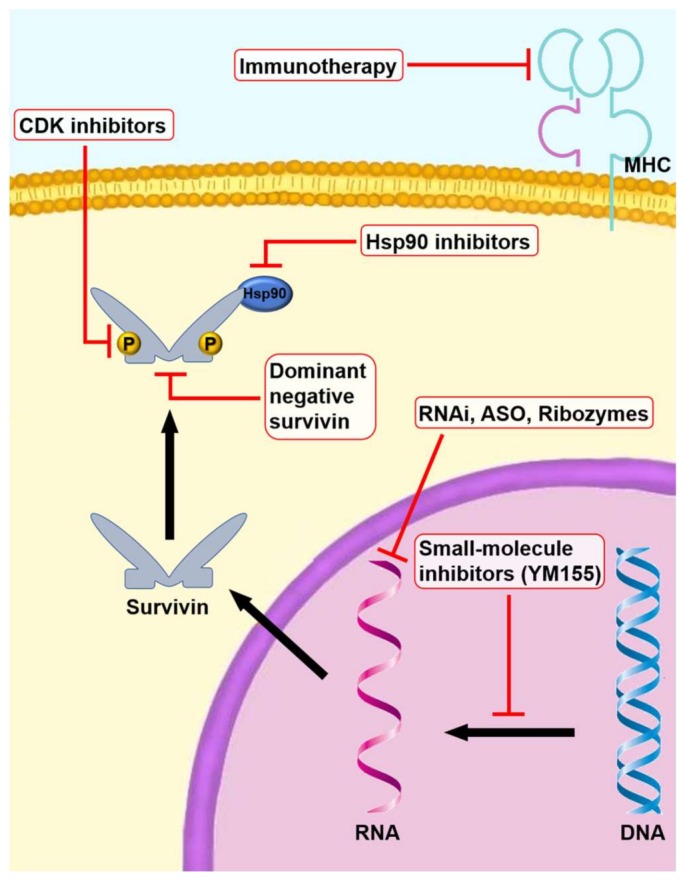
Targeting survivin in cancer therapy. The main classes of therapeutic agents used (RNAi, ASO, SMI, CDK inhibitors, Hsp90 inhibitors, immunotherapy, and gene therapy) are shown in the figure. Red t-bar: inhibition; black-line arrow: translocation of molecules.

Different survivin targeting approaches have been proposed and evaluated for inhibiting tumor growth potential and enhancing tumor response to conventional anti-cancer therapy. These strategies interfere at different levels with survivin expression and include ASO, RNAi, ribozymes, dominant-negative mutants, SMI, vaccines, and adoptive cell therapy (Figure 4). Several clinical trials targeting survivin are being conducted (Table 1) [50].

In recent times, the development of cancer gene therapy was driven by the putative advantages, compared to conventional treatments, of selectively targeting the altered genes in cancer cells. To date, cancer gene therapy research is limited to a few targets and specific tumor types and the investigations regarding survivin in SCC are limited to preclinical models. Conditionally replicative recombinant adenoviral vectors encoding dominant-negative survivin mutants have been successfully used to elicit spontaneous and enhanced drug-induced apoptosis in cell lines of different tumor origins and in cancer xenograft models in nude mice. However, if we consider SCC, and in particular HNSCC, strategies targeting other genes such as p53 have already completed phase III trials. However, as the survivin gene is transcriptionally repressed by wild-type p53 protein and the loss of wild-type p53 in tumors can lead directly to survivin expression [165,166], the administration of rAd-p53 agents may be considered as an approach that indirectly hits survivin.

Although gene therapy strategies, especially the use of siRNA, continue to have an appeal in the development of new anticancer therapeutics, no RNAi technique targeting survivin has reached the clinical trial phase. Despite survivin knockdown by RNAs resulting in proliferation inhibition, apoptosis, and chemosensitivity enhancement in both in vitro and in vivo models, the hope to use this gene-silencing technique has yet to be realized.

Furthermore, there are several technical and safety-related problems regarding the use of gene therapy in clinical practice, and despite significant investments in cancer gene therapy studies, the results have been disappointing [167,168]. Further development is now directed at healthy host tissues with the aim of decreasing the toxicity of conventional therapies or disrupting the tumor niche [169]. Another strategy concerns the use of gene therapy to transfer genes that augment the immune response against cancer cells [170].

On the other hand, the survivin ASO approach has shifted from preclinical investigations, where ASO treatment resulted in significant and stable protein expression inhibition both in SCC cell line and in xenograft nude mice models, to phase I trials in patients with advanced cancers. LY2181308, a second-generation ASO designed to complementarily bind to human survivin mRNA, showed in two dose-escalation studies a favorable safety profile. Despite the fact that only one patient with esophageal SCC was enrolled and showed limited clinical response, results from both trials are encouraging for the future use of LY2181308 as a complementary therapy. However, phase II/III trials are needed.

The abovementioned strategies are based on specific targeting of survivin. An alternative approach, using SMI, is to interfere with pathways that cause abnormal expression of survivin in cancer cells. Although such approaches are not survivin-specific, they have a good chance of obtaining approved for clinical use in the near future. However, this approach has been recently tested with the use of YM155, a novel SMI that directly affects the survivin gene expression via inhibition of its promoter. YM155 was initially tested in two phase I trials that included patients with HNSCC, NSCLC, and esophageal cancer. The administration was considered safe, although more drug-related toxicities were recorded than with LY2181308. Furthermore, YM155 administration requires a 168-h continuous intravenous infusion, with measurable impact on patient compliance. On the other hand, a slightly better clinical response was reported in subsequent clinical trials (Table 1).

However, one of the most intriguing approaches is the ability to stimulate the immune system to initiate a direct response against the tumor. In fact, the main advantage of cancer immunotherapy is the achievement of higher specificity for malignant cells than with conventional therapeutics, preventing the off-target toxicities and inducing efficient anti-cancer responses. There are a few clinical trial addressing SCC cancers, evaluating the safety and tolerability profile of survivin-2B peptide vaccine therapy for patients with advanced or recurrent SCCs. The results showed the absence of serious toxicities after the subcutaneous or intratumoral administration of the vaccine (Table 1). However, despite the better toxicity profile than YM155 or LY2181308, only marginal immunological and clinical responses were achieved.

Several obstacles still stand in the way of realizing the full potential of cancer immunotherapies, such as the high cellular heterogeneity levels found in tumor and metastatic lesions and the emergence of resistance to single-target treatments [171]. In fact, the current approach of targeting single molecular abnormalities can be considered limited. Recent insight regarding the tumor immune regulation will provide the basis for the development of more potent strategies [172].

The current situation of SCC clinical trials is similar to that of other cancer types, such as breast cancer [173], melanoma [142], and malignant glioma [174]. In fact, several studies demonstrated high levels of survivin in many different cancers and its overexpression is associated with poor prognosis, increased rate of recurrence, and resistance to chemo- and radiotherapy. For this reason, several preclinical and clinical studies have been conducted on solid and hematological malignancies using the same molecules already described in this review or with other therapeutic agents. For example, YM155 was evaluated in phase I/II trials giving results similar to those obtained in SCC patients [141,175,176]. Regarding gynecological tumors, several phase I/II trials have been conducted using LY2181308, but the results obtained were unsatisfactory [177]. Cancer immunotherapy is an attractive strategy for the treatment of existing cancers and survivin-based vaccines have been evaluated in several cancer trials, including malignant gliomas. A phase I study involving patients with recurrent malignant glioma was conducted to test safety, immunogenicity, and clinical effects of SurVaxM, a novel peptide mimic immunotherapeutic vaccine, which has not been tested yet in SCCs [174]. A total of 75% of immunologically evaluable patients developed an immune response, and three patients showed a partial clinical response or stable disease, leading to a phase II trial to evaluate the efficacy in patients with newly diagnosed glioblastoma [174,178].

In conclusion, survivin is a unique member of IAP proteins that is upregulated in many tumor types, but the presence of splice variants and different subcellular pools complicate the understanding of this molecule. At the present state-of-the-art survivin-based treatments for cancer, the favorable results from gene therapy pre-clinical studies did not meet expectations in the clinical trials. This is especially true for SCCs where strategies targeting other genes, such as p53, are closer to real clinical use. ASO and SMI seem to be the closest to being incorporated into clinical regimens as they likely reduce the toxicity produced by conventional chemotherapeutic agents. The anticancer immunotherapy with peptide vaccines remain the most intriguing approach but are also far from any practical clinical application in the immediate future. Combined therapies using survivin-based strategies and concomitant/previous chemotherapy seem to be close to a realistic clinical application in SCCs.

## Figures and Tables

**Table 1:** Clinical trials of survivin-targeting agents. PR = partial response, SD = stable disease, MTD = maximum tolerated dose.

Author	Agent	Phase	Site of Cancer	Results	Ref./ID Code
Talbot et al.	LY2181308	I	HNSCC, esophagus	Effective downregulation of survivin. Dose level of 750 mg.	[131]
Tanioka et al.	LY2181308	I	Esophagus	Grade I/II toxicities. Dose level of 750 mg. No case of SD.	[132]
Tolcher et al.	YM155	I	HNSCC, NSCLC	MTD of 4.8 mg/m^2^. 1 case of NSCLC had minor response.	[140]
Satoh et al.	YM155	I	NSCLC, esophagus	MTD of 8.0 mg/m^2^/day. Grade I/II toxicities.	[141]
Kelly et al.	YM155	I/II	NSCLC	Grade II hematological toxicities. Two cases of PR.	[179] NCT01100931
Giaccone et al.	YM155	II	NSCLC	Grade III toxicities 18.9%. Two cases of PR, 14 cases of SD.	[143]
Honma et al.	Peptide vaccine (2B80-88)	I	Urinary tract	Grade I toxicities in six patients. Two cases of SD.	[180]
Miyazaki et al.	Peptide vaccine (2B80-88)	I	Oral cavity	No cases of toxicity. One case of PR.	[153] UMIN000000976
Tanaka et al.	Peptide vaccine (2B80-88)	I	Urinary tract	Grade I toxicities. Six cases of SD.	[181] UMIN00005859
